# Supplementary material for: Single-cell transcriptomes and whole-brain projections of serotonin neurons in the mouse dorsal and median raphe nuclei
Source: eLife. 2019 Oct 24;8:e49424. doi: 10.7554/eLife.49424 (PMC6812963; doi:10.7554/eLife.49424)
Supplement: Supplementary file 4. — Networks were constructed based on Pearson correlation coefficient (rp) of gene expression across all cells. Genes appear connected if rp >0.4. Edge width represents rp. Nodes are colored according to functional gene categories (Materials and methods). [file elife-49424-supp4.zip › visNetwork.html]

visNetwork


Select by idRac1Tcf25Calm1Cdip1Tax1bp1PsapSlc4a3Lrrc8aSlc25a1Etv1Clstn3Vdac3Gata3Pax5Pou6f2Stx1aSlc3a2Rac3TrhGatad1Vdac1Stx7Scn1bSlc6a4Calm3Vamp2Vdac2NfixKlf5Vti1bAsic2Slc22a17Peg3Glra2ChgaProcMaf1Slc7a4Sub1Slc25a11Npffr1Zbtb20Zeb1Tsc22d1Slc25a46Sox9Zfp36l1OtpSlc18a2Crhr2AlcamSlco3a1Slc38a3Epas1Hsf2Id4Grin1Zscan26Myef2Snap25Tbx15Gata2AppRgs2Zeb2Tcf7l2Slc2a10Slc30a9Fgf10FosbSlc10a4GlrbHcrtr1Bcl11aRetSlc5a7Prrx1GabreGabrqTmf1Bhlhe41PspnSlc25a4Slc25a36Cdh13Nr2f2CnbpClcn4Npbwr1ArhgdibFosRorbSlc12a4VtnNfe2l1Cysltr2Kcnh8Chl1Slc6a1Slc7a3Slc39a6Clstn1Ncam1Cx3cl1Nfatc1Runx2Trpc4apPbx3Gadd45gip1Piezo2Slc35g3Snai2PhbAqp8Gad2UncxPcdh8Cacna1eSlc13a1Adgrg6Oxgr1Clic5Hif3aPnocNcam2Gpr35Dscaml1Chrm1Gpr151NpbChrm2Lyl1Rxfp1Calm2Alx4Pcdh10Adra1bHtr1aIrx3PdynNpy2rMyrflSlc39a5Hcrtr2Pcdh7Cntn1Arf1Slc39a1Slc7a2Pcdhb13Hes5Slc29a1Chrna4Kcnk9Gpr4Htr1bSlc8a1En1Klf13Rspo3Adgrl2Slc25a3Zfp706Gpr17CalcrlCcr9Slc17a3Wnt4Grm7Cxcl12Gpr88Sox14Cacng3Sox10Gad1Nhlh1Hrh3Pou3f3Adra1aGpr152Adcyap1Lpar1Pcdh11xIl16InhbeSox21Slc30a2Scn2bZfp978CopaMadcam1Pth2rCdh18Pcdh19Htr1dNrxn3Zfp872Slc18a3Slc17a8Robo1Pcdhac2Olig2Clstn2Tox2PrebJundNpySiglec1Hnf1aProkr1Olig1Gjc3LsampSlc48a1Kcnk16Pou3f1Zfp536Cnr1Cd47Foxa1Npsr1OxtrGpr139Dmrta2Slc39a1.psAdrb1Zfp985Nr3c1P2rx2MafRarres2ClcnkaGjc2Il33Sall1Nhlh2Slc52a3Unc5dGpr119OpcmlMkxZfp771Slc13a4Gpr101Glra1Mcoln1Trpc5Htr7Htr2cItgalAregKcns1Egr2Slc35g2Grm3Zfp981Slc46a2Slc25a41Ackr2CrhChrm5Fpr1Grin2cF2Slc10a5PrlhrPcdh15Wif1AplnTacr3GhrhCdh8Sox1Gpr37l1Slc7a8Itgb1Slc25a54Satb2Scn5aPparaChrm3FevSlc11a1Tgif1Klf1GhrlWnt5aPtgdr2Foxh1Csde1Slc51aGria2Zglp1Slc25a22ToxSlco1c1Foxp2Tcf15EdaItga2Slc25a48Grin2aCeacam20Slc5a12Pou3f2JunEbf1Sox6Efna4Adgrf3Itga4Avpr1aGlra4Kcnk15Chrna10Grm6Slc22a1Celsr1GnrhrTcf24Gm9772Mc5rZfp853Zfp819Taar1Ceacam3Grin3bGm6871Zfp988Slc3a1Gm14393Trp63Il22Cxcl17Gm5767Prdm12Bmp6Kcnk7Slc32a1Slc1a7VegfcEgr3Gm2381Slc22a6Slc6a20aSlamf1Sox2Obox6CrxosGpr25Anxa1Il1rnP2ry13Gm14401Hoxa2Arid3cPax8Neurod4Ntf3Trpm1Tbx2Pcdh12Cd28LhbQrfpHey2Lmx1aMef2bOnecut3Sox15Creb5Slc30a3Hvcn1BocCgaAdora2bHtr3aRxfp2PtafrZfp831Zfp352Ccl2Six5Adgrf2Adgrg3Slc2a2Tbxa2rFzd5Lhx3Clic6Gpr63Sox3Tnfsf13Rfx4Il5Cxcr4Adgre1Cct3RtcbUba1Ndufa11Ndufb2Puf60Mien1AdprhClptm1Tomm40Atp5dPpp5cGlg1Btbd2Pld3Rabac1Dusp3Dgcr6CpRad23aEno2Coro1cAp1s1Map2k1SgtaSyt5Wdr1Prdx2PrkacaPsmd4CsReep5Ergic3NapaAtp5g1Atp6v1b2AampCrip1Pgrmc1Aplp1P4htmHap1Psmd2Abhd16aPpp2r1aBcl2l1Ccdc124CtsdRplp1Abhd8Srp14SardhFxyd5Snap47Rps25Tusc2Nedd8Pgam1Ndufs2Pea15aClip3Chp1Dnajc7CacybpNdufa2Pam16Cox6cTsg101Tppp3Gorasp2Npdc1Ube2iGdi1Steap2Med28Sqstm1DstnH2afyCastor2Atp5eNdufa1Atxn10Psmd3AldocRpl19Timp2Cox7cPitpnaHspb9Gdap1l1Rrp7aCyb5r3Psmb6Kif3aMaptX6330403K07RikZfyve27Sult4a1Atp6ap1Mdh2Atp6v1e1Atp6v0a1Sec14l4Fkbp8Cdc37Pcmt1Crybg1Serinc1Atp2b1Ndufa12Sar1aVsirActr2Uqcr11DdcPhlda1Prmt2Fzr1Sumo3Hint1Mdh1Ppp2caHnrnpabCanxArf5Rps27aRnf187Pdia6Pik3cgAgr2Prkar1aTmem132eCygbRabep1BlmhNxnAlox8Krt19AclyFkbp3Klhdc2SlirpAtl1Tmx1Actr10Srsf5Tc2nGdi2Rgs6WarsHsp90aa1Fdft1Klc1Atp5mplPxdc1Ssr1Habp4CtslTxndc15UqcrbHnrnpkSdhaNdufs6HmgcrScamp1Psmd6Arhgef3NischSpcs1CtsbTktMtmr6Trim35DmtnSucla2Gpc5Dad1Psme1Tgm1CidebSema5aYwhazGtse1St13Samm50Ndufa6Twf1Smdt1Pmm1Aco2Glyr1RogdiApodGrinaLy6hMapk8ip2Yaf2Cd200Rrn3Tmem114TfgGsk3bFstl1Ndufb4Ap2m1Eif4a2Atp5jAtp5oSod1Tuba1bTmbim6Faim2Lima1Ndufa5BsgAcheTmem176aPrphSynj2Acat2Pla2g7Hsp90ab1Enpp5Mrps18aMtch1Abcg1Ndufv3Epb41l3Emilin2Yipf4Ndufv2Mrpl28Dusp1Uqcc2Nudt3Cox7a2lSyt4Mapre2Pfdn6Bin1Csnk2bAtp6v1g2ImpactNdfip1Gnl1Mal2Ppp2r2bPpicTxnl1NarsDctn4Rps14Camk2aDdb1Zfand5Rtn3Otub1Ppp2r5bAipSf3b2Rab1bGalLrp5Arl2Plcb3Stip1Prdx3Gfra1ArhgdiaPcyt2TollipMaged1Got1Ndufb8Actr1aGnl3lMaged2Rps24Jph3Ube3aCd63Cdk2Rps26Rnf41Atp5bDctn2Atp5a1AvilUsp33Clns1aPaip1HrasTktl2Tm9sf2Rbfox3GaaNptx1Cct8HprtUqcrc1St8sia2Nrp1Sec61a2Prkar1bCplx2Cops5Eef1b2Ndufs1Hspd1Ndufb3Inpp4aPlekhb2Bard1Tuba4aDnajb2PtprnItm2cPsmd1Serpine2Ndufa10Stk25Klhl30PamLypd1Ubxn4PtprcTmem9Arl8aAdipor1Fam129aDcaf8Adcy10Nmt2Rgs5PterArpc5lYme1l1PrkcqStxbp1Ak1Dnm1FcnbBC005624Usp20Ndufa8X1700019N19RikEmc4Nop10Syt13Pdia3Lrp4Mtch2Rasgrp1Prom2Nop56Idh3bPcsk2Nsfl1cStmn2Zdbf2GnasAtp9aPsma7Stmn3Map1lc3aGgt7Epb41l1Ndrg3Rpn2Ndufb5Wwtr1PpidKcnab1Hsd3b1Ampd2S100a11X4933434E20RikCalb1Ube2j1Txn1Dnaja1Bag1Dctn3VcpCltaRps6Pde4bTnfrsf8Tmem59Ndufs5Pithd1Akr1a1CapzbPla2g2eVwa5b1Pink1Sema3cHpcaPtp4a2YarsNcdnNudcCdk14Padi2Acot7Cdk5Tomm7Fbxo44Mfn2TprglPrkczFam213bGnb1Sdf4Lrpap1Crmp1Nsg1Snx17Ociad1SgcbMapre3Fam114a1Uchl1PdgfraEpha5Rufy3Wsb2Rimbp2RanNipsnap2Cct6aAnapc5Anxa3Pde6bCitTesX2210016L21RikOasl2Rpl6Erp29Arpc1bNdufa4PompHsph1Fam3cCul1AsnsDync1i1Ccdc136Abcg2Tmem176bCbx3Cct7Copg1Rpn1Mlf2Cops7aGabarapl1Fam234bGolt1bLdhbBcat1Cpne9Camk1CmasNecap1PianpCd9Prmt8Fkbp4Pdcd5Csrp3Pcsk6Apba2Plin1Psmc4Ndufc2CdiptAldoaRab6aPpme1Psma1Rgs10Tial1Dctn5Ubfd1Uqcrc2Anks4bMki67CaskBC051019Ndufb11Cdk16Usp11CttnSypPqbp1ErasMagee1Cox7bSerpina7Acsl4Pak3NonoAp1s2Tktl1Zcchc18Lamp1CoprsSarafSlit2Fgfr1Got2Dnaja2Itfg1TecrCog4Cdyl2Mt3PllpArl2bpCiapin1Polr2cCox4i1Necab2Rab3aDctn1Has3Vps4aKarsAarsTrim29OafAbhd12Arcn1Fez1Pin1Nedd4Gcnt3Tent5aTmprss5PkmRcn2Tspan3Cox7a2Impg1Gsta4MlipRasgrf1Snx1Dync1li1Armc8Cspg5Rab6bAcppGnai2NckipsdSbsponPdxkFbxo21Cspg4CamkvPebp1Fkbp1aEif3lCntnap3Podxl2MgllMzt1Clasp2Eri3Fa2hHacd3Sfxn5SprGucy1a1Gde1Ndufb7Ppp3r1Srsf2NsfHsdl1Chmp7Nipsnap1InaWbp2CmipPja1Cops8Atp5hPpm1hDbn1Tmx4B3galt1Gna11Cpne7Rps29Dapk3Eef2Dpp4Lars2Spcs2Oaz1TpbgAnkrd11Tsen34Mboat7Ndufa3Isg15Wdr18Krt25Plppr3Cox8aPip4p1Tmem59lTmem63bAdamtsl2Lmo1Frmd7Fam69bNdufa13X9530068E07RikSkp1aTmem258Glb1l2Ppp1r11Gpi1Tmem255aAdamts2Atp13a2Tcaf1Zcchc12Cuedc2Cox6b1Tubb4bDpp10C1ql2Trim3Hk1Apbb1Smpd1Ttc29Ndufc1Syn1PldiRsrp1Galnt6Ldlrap1Chst7Actr1bEnpp1Ctbp1Arhgef4Gch1Cldn11Kctd8Atp8a1Cd81Ppp1r16bTgm2Smim14Ccdc92Igsf8AW551984Galnt18Snx10Egr1Usp5Reep2EnsaAtp5j2Atp5lMrps34Txndc5Asb17Rps21SiglecfTimm8bAdrm1Atp6v1g1Prune2Cdc123Rpl22l1Pcsk1nClnkAtp6v0e2Micu3Tspyl4Ccdc27PtpaAjap1Tmem246Stub1Trmt9bStrip2Dusp26RalylAlg2Nhsl1Rhbdd2Plcb4GnazDisp2Tph1Flywch1Sema3dNxph4Pacsin1Ppp1caWdr47Pitpnc1Arglu1Os9Plppr2X1110051M20RikOaz2St8sia4Scamp5Eif4hAgbl2Tex264Atp1a3Rps19Ramp3Vps41Atp1b2Ssr2SelenowCox6a1KirrelMyripAbca8aRpl37Mcm3Ndufa7PigsS100a10Elmod1SvopCdrt4Crtac1FcmrUsp22Klhl14Sem1Asb4SelenokHectd4Bex2Wfdc12EgflamDpy19l1X4930426D05RikD17H6S53ETmem130Sorcs1Fbxl7Rpl7Clrn1OtosFam19a2X2900011O08RikCdc25cSnhg11Tent5cTram1l1Rps2Tceal3Swi5Phf11aSerpinb1aShbUqcrqS1pr1Rpl18aCirbpTlr9Fam186aX2410002F23RikC1ql1X1700101I19RikSlitrk6Nme9Stxbp6Rpl37aRbp1Cox17Kctd4Lyrm4Tmem215Mapk10Mettl21eCcer1Cd24aRpl9Gap43B4gat1Dynlrb1Baiap3Tspyl1Slfnl1CltbTctex1d4Rps8A4galtUsp14Gm8566Palm3Arxes2Gm7075Bcl11bAnkrd46Ctxn1Prex2Frmpd4Lrp1bRetreg2Dcaf7Krt8Rps23Cdc42ep1TecrlClip1C1ql3Arhgap15Rpl36alTmsb4xBC030500Tmx2OpalinX4933406M09RikHtr5bTmem150cTrhdeFtl1Scg2Vwc2Atp5kSpata31d1aGja1Hs3st1Cited1Plcb1NcaldYwhagCbr1Wdfy4Arf3ImmtCemipAtp6v1aD930048N14RikMap1bSae1Reep1PrkcbGas1Sv2bFesMllt11Ndfip2Rab4bTnfaip6SmagpEif3kPde5aTmem179Olfr279X1810037I17RikSpock3Cthrc1Atpif1Sytl5SetFam19a5Tmem158Nyap2Them7Fut9Nap1l5Zcchc24CopeElobRnf181Car10Cfl1Spock1Adap1Tgoln1Cd248Chn1Fkbp2TsnaxGm10015Rpl38SpopGapdhRpl36Camk2bGstm1Sez6lTspan7Gpc6Tubb2aCcser2Fcer1gCol25a1X9630013D21RikNap1l4Pde1aNrsn2Ppp2r5dUqcr10Flot1Ndufs8Ntng1Grb2Psma3Cend1Ap2a1Gm8603Pantr1Rnf14Rpl35aMarf1Gstp1Trim55Tmem263Rpl26Copz1Rrp1HaghlMcrip1Ddhd2NacaTmem151aCox5bDpp6Tac1Rps17Rpl34Erbb4Rpl17Cytl1Tubb3DgkkAp3b2Tubb4aNcs1X9430002A10RikActg1Mrpl42Rpl35LdhaLuzp2Rpl27Mapk1Cyp26b1Rps15Eno1Gpx1Uqcrhmt.Rnr2mt.Tqmt.Co2mt.Atp6mt.Nd3mt.Nd4mt.Nd6Gm23054Gm24451Gm25580Gm22743Adamtsl1Wdr6Fxyd6Oas1eOlfr18Bub3AY702103Rplp0Rps28Cbln4BlcapRomo1Gm10248Rps19.ps9Trp53i11SarsPsma5Col28a1X4930589L23RikHist1h3bHist1h2anMarcksPsmb3Ssbp4Eif3gPlpp4Chchd2Gm572Gm16410Rmdn3Rpl7a.ps8Pnmal2Klhl9Ptges3Gm10318Syndig1lRpl23Usmg5Eef1gB3gat3Gng3PpiaTuba1aGrcc10Trpc5osSod3Acot1Gprasp2Hbq1bX9330158H04RikCops9Gm7534Gm10619Spint2Dda1Gm15441Ankub1Ube2nAU015228Itpripl1Fjx1Klhl41RprmDefb47Ly6aGpx4Trbc2Ighg2cRab11bSnord89AI593442Smim1DohhAnkrd65Emc1Hs3st4Gm17546X1810022K09RikEif5aPrnpC7Kdelr2Lipo4Rab7Tmsb10MaeaRpl39Gm14862Gm8019Gm7571Gm14513Gm15216Rpl35.ps1Gm13815Gm6461Gm14497Gm15989Hspd1.ps4Gm6964Gm13509Gm15951Gm14538Gm5912Gm15877Gm16181Gm15466Gm14473Gm13624Selenot.psGm12331Gm13216Gm15653Gm16216Gm13047Gm11996Gm14162Gm11877Gm5395Gm13740Gm15148Gm11687Gm12129Gm15443Gm12900Gm11663Gm16421Gm15367Rpl15.ps1Gm14832Gm5303Gm8648Gm14869Gm11550Gm5402Rpl7a.ps12Gm14706Gm15538Gm12901Gm13676Gm13071Gm13435Gm14248Gm13007Gm13886Rpl21.ps9Gm5832Gm14439Gm12186Gm13508Gm24598Gm23657Gm12406Gm15473Ubl5Gm14617X1700095J07RikCcdc85cPtprtosFoxo6osBB557941MipeposGm11655Far1osDbhosGm12940Gm13684Rpl9.ps3Gm12335Gm14302Rpl30.ps9Arhgap27os3Trp53cor1Gm11872Gm12144X1810059C17RikErich2osSusd5Gm15475X9130024F11RikAtg4a.psA230108P19RikGm13147Gm13376Gm11464CtcflosGm15558Gm12128Rapgef3os2Cers1Gm15788Gm15202D430040D24RikPnpla1osX1500009L16RikTmem250.psPet100Gm22596Gm25981Nat8b.psGm16559Gm15833Gm8428Gm15882Galnt4Gm16577X4930519F09RikGm15795AU015336Gm17199Sfta2Gm3453Rps27Myl6Gm17112Gm17147Gm17203Gm10722Gm9359X1700030C10RikCcdc152Gm17120Gm17334Ak9Tma7X4930471C04RikGimd1Serpina4.ps1Zfp804bGm5967Gm4246Gm20445Gm20421Gm23639Gm20647Gm7584Gm5777Gm20636Rpl41Obox6.ps1Gm6900Tomm20Gm24497X5430401F13RikGm21984Cdkn2dGm5426Gm15801Galntl6B230364G03RikPafah1b1.ps1Pantr2Gm26743Gm26991Gm7928X2310001H17RikGm10822Gm26815Gm26785Gm26781Gm26783Gm26697Gm26846Gm26847Gm26637Gm17322X2310026I22RikB230344G16RikGm26868X1700039M10RikGm8614Gm8048Gm8709Gm26984Rpl24X5830468F06RikX4930524O08RikMir6336Gm3428Gm27477Gm28513B130024G19RikX1700047M11RikGm4850Gm5256Gm7063Gm19029Gm29461X1700063D05RikGm28372X4930568A12RikGm28175Gm7901Gm7560Gm28402Gm7858Gm28055Gm28268X2210011K15RikGm20125Gm37475SnrpnGm38230Gm37144Gm34106Gm37234Gm37975Gm38144Gm36937Gm18432Gm19694Gm38037X2900097C17RikGm37811Gm37981Gm37729Gm37159X1700051O22RikGm37339Gm37537Gm37999Gm37668Gm37670Gm38215Gm6162Gm37061Gm37608Gm37679Gm37466X5730488B01RikGm6140Gm37399X4930578I07RikGm37689Gm10305Gm35570Gm29784X1700008B11RikGm42864Gm42873Gm20568Gm42519Gm43471Gm3716Gm43301Gm43829Gm32158X4933415J04RikGm42627Gm43400Gm42141Gm43508Gm9497X4930520M14RikGm43491Gm42933Gm43720Gm40319Gm5309Gm9771Gm1969Gm43509Gm2623Gm42900Gm42852Gm42811Gm43701Gm43443Gm43173Gm43262Gm42800Gm43948Gm44104Gm44086Gm6443Gm44106Gm44066Gm44271Gm5340Gm44227Gm19252Gm44056Gm5305Gm44552Gm18642Gm45768Gm44705Gm44978Gm6855Gm44759Gm9521Rpl19.ps9Gm44689Gm44741Gm44873Gm45089Gm44652Gm44715Gm30873Gm44626Gm5737Gm31105Gm45473Gm45644Gm45308Gm2033Gm9911Gm45456Gm7619Gm45889Pde2aGm45607Gm45424Gm45756C230057M02RikGm18935Gm48784Gm8171Gpx4.ps2Gm47235Gm49427Gm47233Gm47867Gm18655Gm47047Gm47430Gm47356Gm8031Gm47640Gm5950Gm18119X4930563J15RikGm47894Gm48077Gm47874Gm49376Gm48772Gm19801Gm48324Gm2696Gm48084Gm7940Gm33378Gm34376Gm46332Gm18499Gm5954Gm3333Gm35161Gm47813Gm33312X4930477G07RikGm20063Gm7065Gm47368X1700015C15RikGm33785Gm36500Ndufb1.psGm48140Gm7046Gm7986Gm34307Rpl13.ps2Gm48767Gm7621Gm48488Gm17805Gm41183Gm5461Gm47918Gm48438Gm18760X5430425E15RikGm9502Gm49195Gm8705Gm48909Gm19016Gm49017Gm17932Gm49209Gm46448Gm49196X4930445N06RikGm8843PdxpGm32405Gm18724CT030190.1AC166832.1AC153140.1CT010565.2AC125199.3AC171202.1AC154408.2AC115120.1CT010433.1CT010490.2CT027991.1Atp5o.1AC154275.2CAAA01141682.2AC132911.2AC144797.1AC154457.2AC154343.2AC159289.1AC134555.1AC118208.1AC127347.1AC134555.2

---

Select by groupAmineReceptorsCAMGABARGlutamateReceptorsGPCRIonChannelNeuropeptideReceptorsReceptorLigandsSignalTransmissionSNARETF

Export as image

---
